# Sclerostin Antibody Mitigates Estrogen Deficiency-Inducted Marrow Lipid Accumulation Assessed by Proton MR Spectroscopy

**DOI:** 10.3389/fendo.2019.00159

**Published:** 2019-03-20

**Authors:** Shaojun Li, Bingcang Huang, Bo Jiang, Mingjun Gu, Xiaodan Yang, Ying Yin

**Affiliations:** ^1^Department of Radiology, The Second Military Medical University Affiliated Gongli Hospital, Shanghai, China; ^2^Department of Endocrinology, The Second Military Medical University Affiliated Gongli Hospital, Shanghai, China

**Keywords:** sclerostin, bone marrow, osteoporosis, estrogen deficiency, MR spectroscopy

## Abstract

Sclerostin knock-out mice or sclerostin antibody (Scl-Ab) treated wild-type mice displayed decreased marrow adiposity. But the effects of Scl-Ab on estrogen deficiency-induced marrow fat expansion remain elusive. In this work, 45 female New Zealand rabbits were equally divided into sham-operation, ovariectomy controls, and ovariectomy treated with Scl-Ab for 5 months. MR spectroscopy was performed to longitudinally assess marrow fat fraction at baseline conditions, 2.5 and 5 months post-operatively, respectively. We evaluated bone mineral density (BMD), bone structural parameters, serum bone biomarkers, and quantitative parameters of marrow adipocytes. Ovariectomized rabbits markedly exhibited expansion of marrow fat in a time-dependent manner, with a variation of marrow fat fraction (+17.8%) at 2.5 months relative to baseline and it was maintained until 5 months (+30.4%, all *P* < 0.001), which was accompanied by diminished BMD and deterioration of trabecular microstructure. Compared to sham controls, adipocyte mean diameter, adipocyte density and adipocytes area percentage was increased by 42.9, 68.3, and 108.6% in ovariectomized rabbits, respectively. Scl-Ab treatment increased serum bone formation marker and alleviated the ovariectomy escalation of serum bone resorption marker. It remarkably lessened the ovariectomy-mediated deterioration of BMD, and morphometric characteristics of trabecular bone. Marrow fat fraction was decreased significantly with Scl-Ab to levels matching that of sham-operated controls and correlated positively with reductions in adipocyte mean diameter, percentage adipocyte volume per marrow volume, and adipocyte density. Taken together, early Scl-Ab treatment reverts marrow fat expansion seen in ovariectomized rabbits in addition to having a beneficial effect on bone mass and microstructural properties.

## Introduction

Osteoporosis remains largely underdiagnosed and undertreated, even after the first fracture has occurred. Based on the currently available evidence, bisphosphonates are generally recommended as the first line pharmacological agents for osteoporosis (1). Due to progresses in understanding the mechanisms and the causes of bone fragility, novel and promising therapeutic targets for osteoporosis have been identified (2). Studies in both animal models and human of sclerosteosis and van Buchem disease have demonstrated that sclerostin plays an important role in regulating bone metabolism (3–5). Sclerostin is now recognized as a new target for the treatment of patients with osteoporosis and other skeletal disorders. Sclerosteosis and van Buchem disease result in generalized high bone mass due to overactive osteoblast activity (6, 7). Subsequent studies have indicated that monoclonal antibodies to sclerostin such as romosozumab have been used in preclinical and clinical studies of osteoporosis with beneficial outcomes for bone mass and microstructure, and fractures risk reduction (4, 5, 8). Previous studies demonstrated that estrogen-deficient women had higher sclerostin serum levels (9), and high serum levels of sclerostin were associated with increased fracture risk, particularly in populations with lower bone mass (10). However, the exact source of sclerostin within the bone marrow microenvironment under pathophysiologic conditions, the mechanisms by which sclerostin regulates the activity of osteoblasts and osteoclasts, and its autocrine effects on osteocytes remain elusive (11, 12).

Marrow adipose tissue is a fat depot with unique features distinguishing it from the better characterized extramedullary sites (13). Much attention has been paid to marrow adipocyte formation and its physiological and pathophysiological implications for skeletal remodeling. Enhanced marrow adiposity formation is found well-related with bone loss at different skeletal sites in most conditions such as aging or menopause (14–16). Marrow fat content increases approximately 7% per decade in the lumbar spine from 30% at age 30 to >60% at the age of 80 years in healthy adults (17, 18). Experimental and clinical evidence supports that an increase in marrow adipogenesis from mesenchymal stem cells at the expense of osteoblastogenesis, contributes to the etiology of osteoporosis (16, 19, 20). Increased marrow adiposity has been shown to be an independent indicator of bone deterioration and fractures risk (19), and reduced marrow adiposity is considered as a potential therapeutic target (20, 21).

Sclerostin antibody (Scl-Ab) acts to restore bone mass, trabecular microstructure and strength in ovary-intact and ovariectomized (OVX) rats (4, 5, 22–24). *Ex vivo*, sclerostin can induce adipogenesis in 3T3-L1 cells, mouse or human bone marrow-derived mesenchymal stromal cells. Fortunately, sclerostin knock-out mice or Scl-Ab treated wild-type mice showed reductions in marrow adipocyte number and size (25). But the efficacy of Scl-Ab for enhanced skeletal health is not yet fully understood. Particularly, the effect of Scl-Ab on estrogen deficiency induction of marrow fat expansion has not been tested *in vivo*. In this work, we aimed to address this question. We therefore hypothesized that sclerostin antibody mitigates OVX-inducted marrow fat accumulation and bone mass loss. To test this hypothesis, in this longitudinal study we detected the sequential effects of sclerostin antibody on marrow fat fraction by using proton MR spectroscopy in OVX rabbits over a 5 month period.

## Materials and Methods

### Experiment Protocol

This study was carried out in accordance with the recommendations of the Association for Assessment and Accreditation of Laboratory Animal Care International. The protocol was approved by the Second Military Medical University Affiliated Gongli Hospital of Institutional Animal Care and Use Committee (approval no., 2017-GL081).

Forty-five skeletally mature, 24-week-old female New Zealand White rabbits were purchased from Silaike Experimental Animals Co., Ltd. (Shanghai, China) and were allowed to acclimatize with free access to water and a standard commercial rabbit chow (containing 0.8% calcium and 0.5% phosphate) for 2 weeks before use. Throughout the experimental study, the rabbits were kept individually in cages under a 12 h light/dark cycle at room temperature. The rabbits were divided into the sham-operation + vehicle, OVX + vehicle and OVX + Scl-Ab groups (*n* = 15/per group). For the surgery procedure, anesthesia was induced with intravenous injection of 3% sodium pentobarbital (1 ml/kg) through the marginal ear vein. A midline incision was distally made from the umbilicus for 4–5 cm. The linea alba and peritoneal tissue below were incised, thereby protecting the intestines. For the OVX group, the bilateral ovaries were located, and the ovarian vessels were ligated. The ovaries were removed with their ligamentous attachment on the uterine horn, while in the case of the sham-operated rabbits, the ovaries were exteriorized and then placed back into the abdominal cavity intact. The linea alba and abdominal muscles were then closed followed by the skin using an absorbable suture. For postoperative analgesia, buprenorphine was given intramuscularly at a dose of 0.043 mg/kg for 3 days. All rabbits were treated starting the fourth day after operation. OVX + Scl-Ab group received Scl-Ab at 13 mg/kg, twice weekly by subcutaneous injection for 5 months. The SclAb dose used in the study was based on previous reports (22, 26). Saline injections were administered to sham and OVX controls with the same frequency and period. Each rabbit was weighed every week during the experiments. Dosages were adjusted weekly based on body weight.

At the end of the experiment (3 days after the final injection of Scl-Ab), blood samples were collected from auricular veins. The samples were centrifuged at 3000 rpm, at 4°C for 10 min and plasma samples were collected for further biomarker analysis. After that, uteruses were directly removed and weighed after the animals were euthanized by an overdose of sodium pentobarbital intravenously. Then we collected the bone specimens. Dual-energy x-ray absorptiometry (DXA) was performed to measure the *in vitro* bone mineral density (BMD) at the isolated left whole femur. Following BMD measurements, left femora were decalcified for histopathological examination and the right femora were collected for a micro-CT analysis.

### Marrow Fat Fraction Measurements

The rabbits were ventrally positioned with hind limbs separated from the trunk to scan the left femur at baseline conditions, 2.5 and 5 months under general anesthesia as mentioned above. *In vivo* MR spectroscopy data were collected using a 3-T instrument (Siemens Trio, Siemens Medical Systems, Erlangen, Germany) with the integrated body coil for signal transmission and a quadrate knee array for signal reception. Before the acquisition of MR spectroscopy data, MR imaging with sagittal, coronal, and axial scout T2-weighted fast spin echo sequence at the left femur were performed to prescribe the spectral acquisition box.

After imaging, single-voxel MR spectroscopy data were acquired at the distal femur using a single-voxel point-resolved spectroscopy pulse sequence (repetition time = 5,000 ms, echo time = 30 ms, 64 averages without water suppression, number of data points = 1,024, voxel size = 6 × 6 × 14 mm3, receiver bandwidth = 2,000 Hz). For each voxel placement, automated optimization of gradient shimming was performed. The average duration of the overall acquisition for the localizer sequence and the bone marrow MR spectroscopy including shimming was about 5 min.

Spectra were processed in the time domain with jMRUI software based on an Accurate, Robust, and Efficient Spectral fitting (AMARES) algorithm. Preprocessing included calibration according to the main–(CH_2_)n–peak (1.30 ppm) and zero order automatic phasing. No constraints were applied on line widths, and peak frequencies were adjusted to lie within 0.1 ppm according to their theoretical chemical shifts. Five peaks including the water peak at 4.7 ppm and four lipid peaks emerged at 0.9, 1.3, 2.3, and 5.2 ppm were quantified, referred to the assignment in previous studies (16). After processing, the amplitudes of the lipid and water peaks were quantified. Marrow fat content is calculated as fat fraction (FF) = [I_lipids_/(I_lipids_ + I_water_)] × 100%, where I_lipids_ is the sum of the area amplitudes of the resonances (locations at 0.9, 1.3, 2.3, and 5.2 ppm) and I_water_ is the area amplitude of H_2_O resonance (19).

### Measurements of Serum Sclerostin and Bone Biomarkers

Serum biomarkers of bone turnover including bone formation marker C-telepeptide type I collagen (CTX-I) and bone resorption marker bone-specific alkaline phosphatase (BSAP) (CUSABIO, Wuhan, China), and serum levels of sclerostin (Boster Biological Technology, Wuhan, China) were quantified using enzyme-linked immunosorbent assay (ELISA) kits, according to the manufacturers' instructions.

### BMD Measurements

Area BMD at the L5 vertebrae and left femur was measured by a Hologic Discovery Wi DXA Scanner (Hologic Inc., Bedford, MA, USA; version 12.7); specific software for small animal-scanning mode was used as described elsewhere (27). BMD value was automatically calculated using the bone mineral content of the measured area.

### Micro-CT Analysis

A cone-beam X-ray micro-CT system (Healthcare Explore Locus, GE Medical Systems, Milwaukee, USA) was used to obtain CT images of the right femur using the following parameters: X-ray tube voltage = 80 kV; anode current = 80 mA; shutter speed = 3000 ms; binning factor = 2; angle of increment = 0.5°; and spatial resolution = 14 μm/voxel. Three-dimensional images were reconstructed in 1024 × 1024-pixel matrices and analyzed using the Microview v2.1.2 software program. Cancellous bones at the distal femoral metaphysis were evaluated beginning at 0.5 mm proximal to the most proximal point along the boundary of the growth cartilage with the metaphysis. Bone volume/total volume (BV/TV), trabecular thickness (Tb.Th), trabecular number (Tb.N), trabecular separation (Tb.Sp), and structure model index (SMI) were determined.

### Histopathological Evaluation

Briefly, the left femurs were fixed in 10% buffered formalin for 2 days and then decalcified with 10% ethylene diamine tetraacetic acid for 5 weeks, and dehydrated with concentrated ethanol, and embedded in paraffin wax after being washed with xylene; they then were cut into 5 μm sections along the coronal plane of the distal femora. For quantifying marrow adipocytes, sections were subjected to routine hematoxylin and eosin (H&E) staining. Five fields from a single section with a magnification of 400× per sample were randomly selected for evaluation using a light microscope (Nikon AZ100; Nikon, Tokyo, Japan) and image J software (NIH) to calculating adipocyte mean diameter, percentage adipocyte volume per marrow volume, and adipocyte density [adipocyte number per unit bone marrow area (excluding the bone trabeculae) in the analyzed fields], as described previously (28). The mean value of quantitative parameters of marrow adipocytes was taken for statistical analysis from all fields measured for each rabbit.

### Statistical Analysis

Analysis was performed with SPSS Advanced Statistics (version 18). Data are expressed as the mean ± standard deviation. Normal distribution of data in each group was confirmed by a Shapiro-Wilk test. Differences in multiple-time point measurements over time were tested for FF data using repeated-measures analysis of variance (ANOVA). Bonferroni *post-hoc* test was used to detect the differences between groups at each time point. Differences in other studied parameters were analyzed using one-way ANOVA with the Bonferroni *post-hoc* test. All statistical tests were two-tailed, and a *P*-value < 0.05 was considered to be significant.

## Results

### General Outcomes

**Figure 1:**
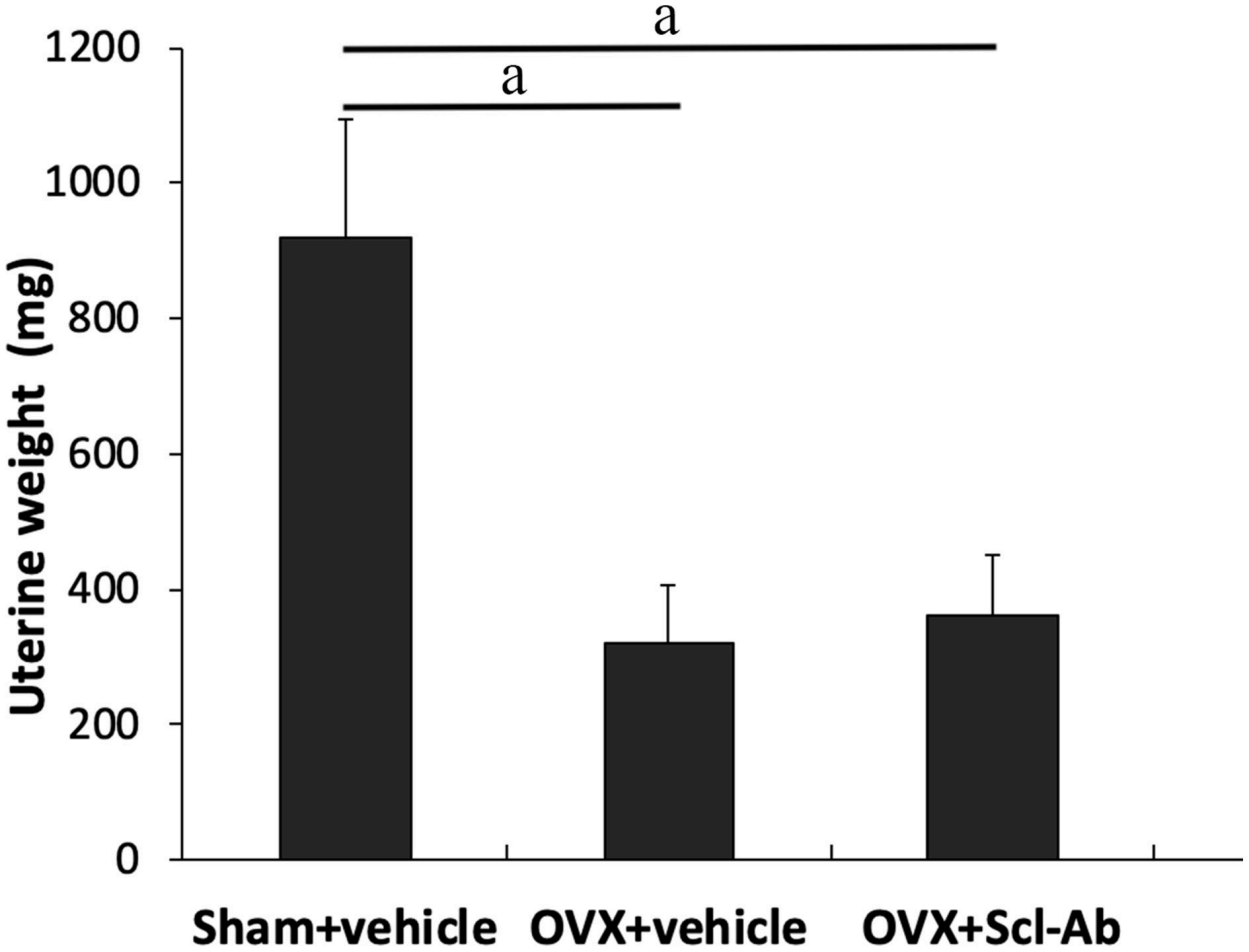
Effects of OVX and sclerostin antibody on uterine wet weight Data are expressed as mean ± SD (*n* = 14, 12, and 13 rabbits in Sham + vehicle, OVX + vehicle and OVX + Scl-Ab, respectively). OVX, ovariectomy; Scl-Ab, sclerostin antibody; Sham, sham-operation. ^a^*P* < 0.001 comparison with the sham controls analyzed by one-way ANOVA with the Bonferroni *post-hoc* test. No significant difference in uterine wet weight was observed between the sham controls and OVX + Scl-Ab.

38 rabbits (38/45, 84.4%) completed the whole study, whereas 6 rabbits (1, 3, and 2 rabbits in sham controls, OVX controls, and Scl-Ab-treated rabbits, respectively) died of an adverse reaction to anesthetics. Scl-Ab treatment did not significantly alter the body weight of OVX rabbits. At the age of 46 weeks, the final mean body weight gain was comparable in the three groups (sham controls, 1.35 ± 0.20 kg; OVX controls, 1.86 ± 0.40 kg; OVX + Scl-Ab, 1.79 ± 0.36 kg; *P* = 0.380). No adverse gastrointestinal effects (diarrhea or vomiting) or surgical complications (mortality, infection, or wound dehiscence) were found. The success of ovariectomy was confirmed in all OVX rabbits by clear atrophy of the uterine horns at necropsy. OVX rabbits had lower uterine weights than the sham controls. Treatment with Scl-Ab did not affect uterine weight decreased in OVX rabbits (Figure 1).

### Changes in Serum Sclerostin and Biomarkers of Bone Turnover

Of note, rabbits receiving Scl-Ab injections showed 31.6% decrease in serum sclerostin concentrations than that of OVX controls at 5 months postoperatively (Table 1). After 5 months, with regard to the levels of serum bone biomarkers, OVX rabbits showed significant increases in bone resorption marker CTX-1, whereas serum bone formation marker BSAP was significantly reduced. Treatment with Scl-Ab significantly lowered the OVX-induced augmentation of serum CTX-I levels and increased serum levels of BSAP.

**Table 1:** Effects of OVX and sclerostin antibody on serum sclerostin and bone biomarkers in the three groups of rabbits.

	**Sham + vehicle (*n* = 14)**	**OVX + vehicle (*n* = 12)**	**OVX + Scl-Ab (*n* = 13)**
Sclerostin (pg/ml)	31.5 ± 5.8	40.9 ± 6.7^a^	33.6 ± 5.5^b^
CTX-I (ng/ml)	31.5 ± 5.8	40.9 ± 6.7^a^	33.6 ± 5.5^b^
BSAP (mIU/ml)	9.8 ± 1.1	7.9 ± 1.2^a^	9.1 ± 1.3^b^

*BSAP, bone-specific alkaline phosphatase; CTX-I, C-telopeptide of type 1 collagen cross-links; OVX, ovariectomy; Scl-Ab, sclerostin antibody; Sham, sham-operation. Data are expressed as mean ± SD*.

a P < 0.05 comparison with the sham controls and

b *P < 0.05 comparison with the OVX controls analyzed by one-way ANOVA with the Bonferroni post-hoc test. No significant differences were observed between the sham controls and OVX + Scl-Ab*.

### Effects of Scl-Ab on BMD and Trabecular Microarchitecture

The vehicle-treated OVX rabbits exhibited mild responses to the estrogen deficiency-mediated loss of bone mass of the lumbar spine and femur. Likewise, micro-CT images showed trabecular microstructure deteriorations of the femur in the OVX controls, exhibiting significant decreases in BV/TV, Tb.Th, and Tb.N but significant increases in Tb.Sp and SMI. Scl-Ab treatment prevented the OVX-induced losses of BMD and morphometric properties of trabecular bone, exhibiting significant increases in BV/TV, Tb.Th, and Tb.N, as well as bone mass. The ovariectomy escalation of Tb.Sp and SMI was also attenuated after Scl-Ab administration (Table 2).

**Table 2:** Effects of OVX and sclerostin antibody on bone mass and microstructure in the three groups of rabbits.

	**Sham + vehicle (*n* = 14)**	**OVX + vehicle (*n* = 12)**	**OVX + Scl-Ab (*n* = 13)**
Femur BMD (*g*/*cm*^2^)	358 ± 35	326 ± 28^a^	350 ± 37^b^
L5 vertebrae BMD (*g*/*cm*^2^)	298 ± 39	260 ± 35^a^	286 ± 33^b^
BV/TV (%)	32.1 ± 5.3	23.3 ± 4.5^a^	30.1 ± 5.0^b^
Tb.Sp (μ*m*)	220 ± 61	389 ± 80^a^	262 ± 72^b^
Tb.Th (μ*m*)	125 ± 21	103 ± 19^a^	119 ± 17^b^
Tb.N (1/mm)	3.01 ± 0.69	1.98 ± 0.58^a^	2.78 ± 0.62^b^
SMI	0.82 ± 0.18	1.51 ± 0.35^a^	0.95 ± 0.31^b^

*BMD, bone mineral density; BV/TV, Bone volume/total volume; OVX, ovariectomy; Scl-Ab, sclerostin antibody; Sham, sham-operation; SMI, structure model index; Tb.N, trabecular number; Tb.Sp, trabecular separation; Tb.Th, trabecular thickness. Data are expressed as mean , SD*.

a P < 0.05 comparison with the Sham controls and

b *P < 0.05 comparison with the OVX controls analyzed by one-way ANOVA with the Bonferroni post-hoc test. No significant differences were observed between the sham controls and OVX + Scl-Ab*.

### Scl-Ab Treatment Lowered Marrow Lipid Accumulation in OVX Rabbits

**Figure 2:**
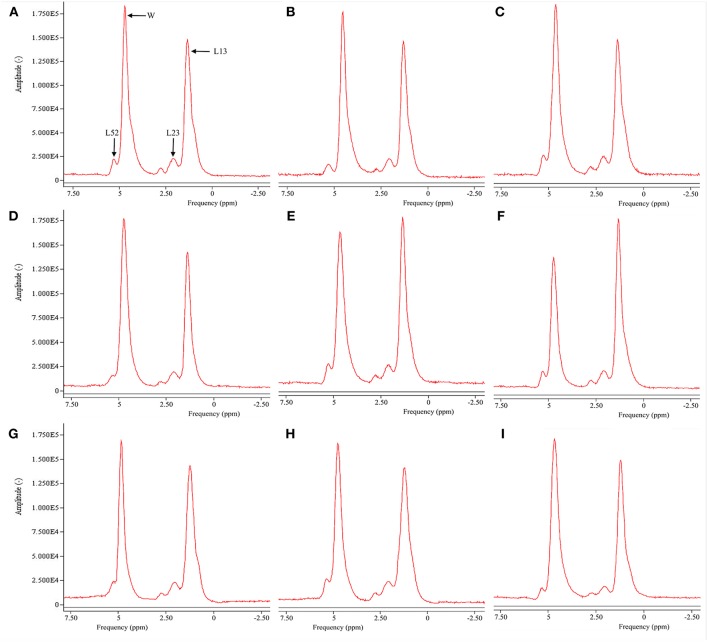
Representative proton MR spectroscopy at the left proximal femur of a rabbit from the SHAM control at baseline conditions, 2.5 and 5 months [**(A)**, FF = 42.9%; **(B)**, FF = 42.5%; **(C)**, FF = 42.7%], OVX control at baseline condition, 2.5 and 5 months [**(D)**, FF = 43.1%; **(E)**, FF = 50.1%; **(F)**, FF = 56.5%, respectively), and Scl-Ab-treated group at baseline condition, 2.5 and 5 months [**(G)**, FF = 43.5%; **(H)**, FF = 43.6%; **(I)**, FF = 44.0%, respectively]. OVX, ovariectomy; Scl-Ab, sclerostin antibody; Sham, sham-operation. Bulk CH_2_ methylene protons (labeled L13 at ~1.3 ppm), the CH_2_ methylene protons α- (L23 at ~2.3 ppm), water (W at ~4.7 ppm), and olefinic protons overlapped with the glycerol CH methine proton (L52 at ~5.20 ppm). CH_3_ methyl protons (0.9 ppm) and bulk CH_2_ methylene protons (1.3 ppm) appeared as a single peak at clinical field strengths (≤3T MRI).

**Figure 3:**
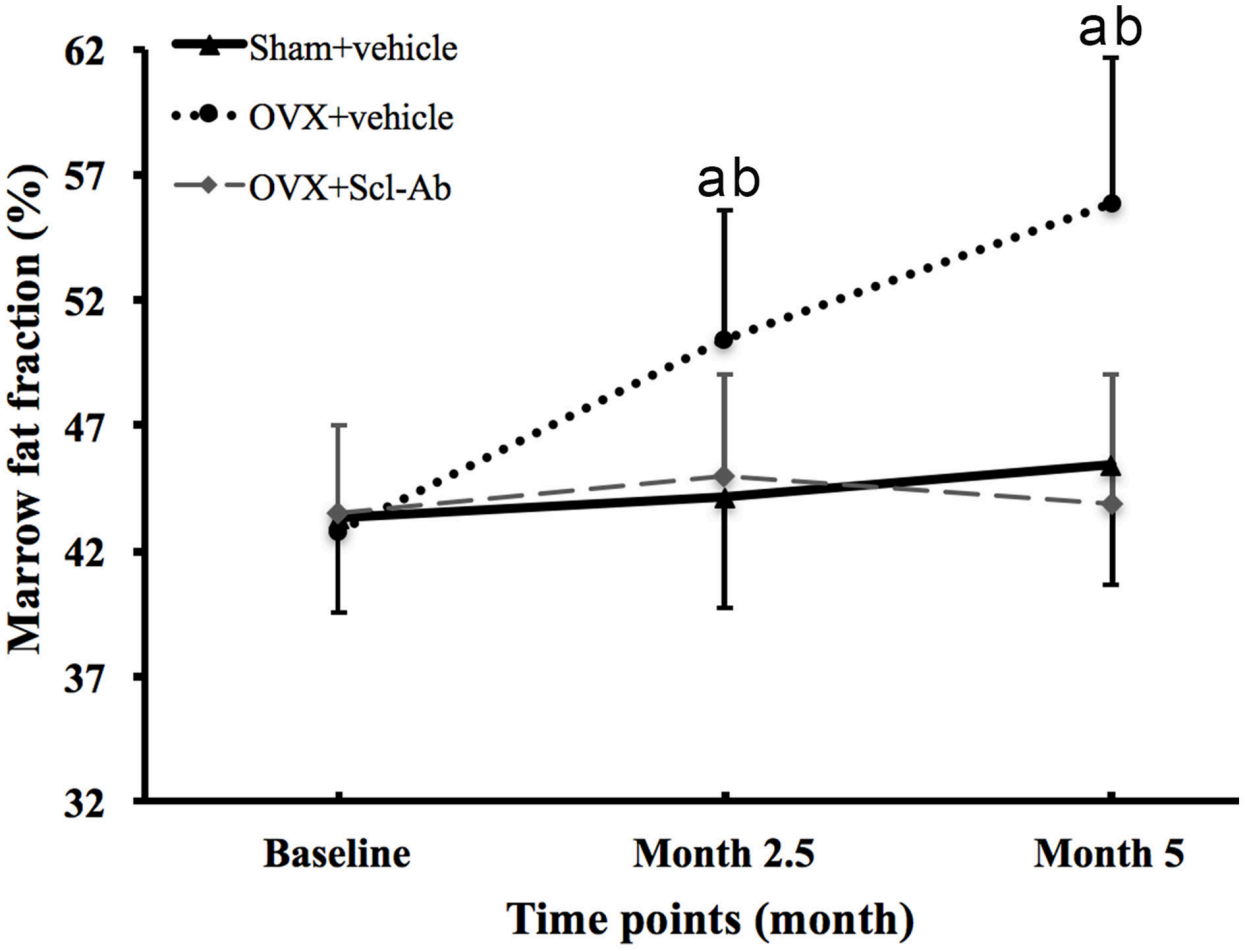
Changes in marrow fat fraction at the femur in all groups at all time points. Data are expressed as mean ± SD. OVX, ovariectomy; Scl-Ab, sclerostin antibody; Sham, sham-operation. ^a^*P* < 0.001 vs. previous time point in OVX + vehicle group. ^b^*P* < 0.01 vs. Sham + vehicle and OVX + Scl-Ab groups at the same time point.

Figure 2 is the representative proton MR spectroscopy at the left distal femur monitored at the various time points. Changes in FF of the distal femur were shown in Figure 3. Relative to the sham controls, FF in the OVX group was increased by 14.3% at 2.5 months and 22.9% at 5 months, respectively. Among the different time points, FF in the OVX controls of 2.5 and 5 months post-operatively was different. No significantly temporal changes in FF were detected in the sham group. Compared to the OVX controls, a significant reduction in bone marrow FF (−10.7% at 2.5 months and −21.3 % at 5 months, respectively, all *P* < 0.001) was seen in the Scl-Ab-treated group. At the end of the experiment, bone marrow FF in the Scl-Ab-treated group was comparable to levels of the sham controls ([43.9 ± 5.1]% vs. [45.4 ± 4.8]%, *P* > 0.05).

**Figure 4:**
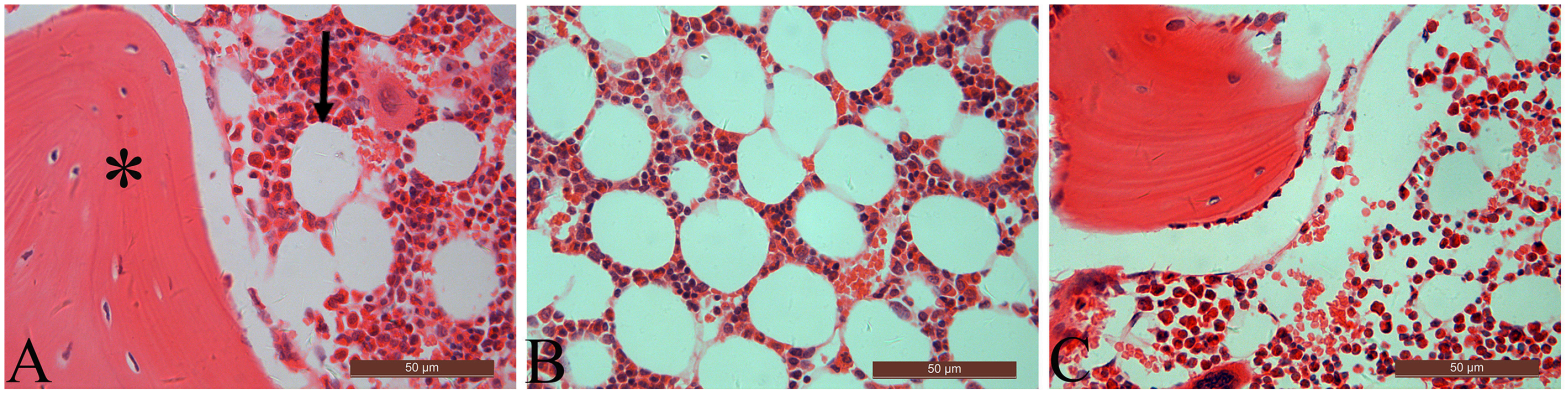
The distal femur stained with hematoxylin and eosin (original magnification ×400). For the sham-operated rabbit **(A)**, abundant trabecular bone (asterisk) in conjunction with few marrow adipocytes (arrow) were embedded in the bone marrow cavity. Marrow fat expansion induced by ovariectomy **(B)** is alleviated with concurrent sclerostin antibody treatment **(C)**.

Table 3 summarizes data of marrow adipocyte measurements. Consistent with the MR spectroscopy results, specimens in the OVX controls showed scanty trabecular bone histopathology in conjunction with abundant marrow fat formation as evident from hematoxylin and eosin staining. Relative to the sham controls, OVX led to an increase in adipocyte density by 68.3%, adipocyte mean diameter by 42.9%, and percentage of adipocyte volume per marrow volume by 108.6%. The severity of OVX-induced bone marrow histopathology was evidently alleviated after Scl-Ab administration (Figure 4).

**Table 3:** Scl-Ab treatment rescued marrow adipocyte accumulation in OVX rabbits.

	**Sham + vehicle (*n* = 14)**	**OVX + vehicle (*n* = 12)**	**OVX + Scl-Ab (*n* = 13)**
Adipocyte mean diameter (μ*m*)	21.2 ± 3.0	30.3 ± 3.5^a^	22.4 ± 3.2^b^
Adipocyte density (1/*mm*^2^)	82 ± 25	138 ± 34^a^	85 ± 23^b^
Percentage of adipocyte area (%)	26.8 ± 8.2	55.9 ± 11.3^a^	27.9 ± 8.0^b^

*OVX, ovariectomy; Scl-Ab, sclerostin antibody; Sham, sham-operation. Data are expressed as mean ± SD*.

a P < 0.001 comparison with the Sham controls and

b *P < 0.001 comparison with the OVX controls analyzed by one-way ANOVA with the Bonferroni post-hoc test. No significant differences were observed between the sham controls and OVX + Scl-Ab*.

## Discussion

Accumulating evidence shows that blocking sclerostin action delays the pathogenesis of various skeletal disorders. Administration with monoclonal antibody romosozumab increases BMD and reduces the risk of fractures in postmenopausal women (29). Treatment with Scl-Ab in an animal model of postmenopausal osteoporosis, not only resulted in complete reversal of estrogen deficiency-induced bone loss, but also further increased bone mass and bone strength to levels greater than those seen in non-OVX controls (30). Similar results were found in a gonad-intact aged male rat model (31), an orchiectomized rat model of male osteoporosis (22), or in female, gonad-intact cynomolgus monkeys (32). Our data were in agreement with the study revealing that sclerostin antibody treatment mitigated the estrogen deficiency-mediated deterioration of bone mass, trabecular microarchitecture, and biomechanical properties (4). Interestingly, Scl-Ab also suppressed bone resorption (22). Thus, Scl-Ab is being considered as the pharmacologic agent with dual properties–promoting bone formation and inhibiting bone resorption.

Estrogen regulates bone turnover and its deficiency stimulates sclerostin expression leading to increase serum sclerostin (9). Treatment with estrogen was associated with a significant reduction in bone sclerostin mRNA levels and with lower mRNA levels of the sclerostin-related protein, sclerostin domain-containing protein 1 (9). Unfortunately, the change in serum levels of sclerostin after sclerostin antibody injection remains elusive. The anti-receptor activator of NF-kappaB ligand antibody denosumab treatment was associated with significant increases in serum sclerostin levels in postmenopausal osteoporosis (33); however, others reported serum sclerostin concentrations was not affected by denosumab or zoledronic acid medication (34). We found that injection with Scl-Ab significantly reduced serum sclerostin concentrations in OVX rabbits at 5 months postoperatively. In experimental animals receiving Scl-Ab, low serum sclerostin concentrations was associated with high bone mass in PTH-administered mice (35) and BMP receptor knockout mice (36).

Marrow adipose is a heterogeneous fat depot whose characteristics differ from the subcutaneous and visceral adipose tissue (37). Clinical and fundamental studies have paid much attention to the complex relationship between marrow lipid accumulation and bone health. Actually, marrow adipose tissue could contribute to deterioration of skeletal integrity via secreting various paracrine factors to influence osteoblasts and /or osteoclasts formation and function (37). Targeting the process of marrow adipogenesis may lead to the development of new therapeutic targets for “fatty bone” and osteoporosis. Higher circulating sclerostin was associated with higher marrow fat content, suggesting that osteocyte activity may also influence marrow fat (38). Sclerostin deficiency lowers marrow adiposity but raises trabecular bone volume (25). Nevertheless, it remains unclear whether sclerostin inhibition can rescue estrogen deficiency-induced marrow adiposity.

A key finding of this work was that, Consistent with the MR spectroscopy-based FF, reduction in adipocyte density, adipocyte mean diameter, and percentage of adipocyte volume per marrow volume also substantiated the phenomenon that early Scl-Ab treatment reverted estrogen deficiency-induced marrow fat expansion. Similarly, Fairfield et al. (25) demonstrated that a decrease of sclerostin *in vivo*, via both sclerostin-knockout and Scl-Ab methods, significantly reduced marrow fat formation. In further agreement with our results, in a study of type I diabetic mouse model, Yee et al. (39) found a significant reduction in marrow adipocyte within the healing fracture callus in Scl-Ab-treated mice, suggesting that Scl-Ab treatment may in this way enhance fracture healing by directly or indirectly lowering marrow fat expansion, independent of its effects on osteoblast maturation or activity. However, in uninjured bones, they found a significant effect of diabetes on the adipocyte number, with no effect of Scl-Ab treatment. These unexpected findings may be attributed to the use of different experimental model and the antibody dose.

Canonical Wnt signaling promotes osteoblastogenesis bone via regulating osteoblast proliferation and differentiation. Sclerostin is predominantly secreted by mature osteocytes that are the most abundant cells in the bone. Sclerostin binds to first propeller of low density lipoprotein receptor related protein 5/6 co-receptors and dampens Wnt and frizzled receptor complex formation, resulting in significant suppression of canonical β-catenin-dependent Wnt signaling in osteoblasts (25, 40, 41). Scl-Ab impacts the Wnt signaling pathway, which in addition to its role in bone homeostasis, also stimulates differentiation of mesenchymal stem cells to osteoblasts by suppressing CCAAT/enhancer-binding protein alpha and peroxisome proliferator-activated receptor gamma (25).

The strength in our work is that, we used a longitudinal experiment design to assess the sequential changes of Scl-Ab on marrow lipid accumulation using MR spectroscopy. The limitations of our study must be acknowledged. First, the dose of Scl-Ab used in this study was based on previous reports (42), which was intended to overcome the potential immune responses to the therapeutic protein, greatly exceeded the dose used in clinical trials. Further studies are required to evaluate the impact of alternative doses on marrow fat expansion. Second, although we performed a long-term temporal design to detect the dynamic changes in the effects of Scl-Ab on marrow adipogenesis, interpretation is challenging for lack of a negative control model that resists marrow lipid accumulation. Finally, since we do not have a separate sham + Scl-Ab group and the *in vitro* evidence such as lacking of dedicated staining for adipocytes like osmium, is inadequate to prove the effect of Scl-Ab on marrow adipocytes, the results could be just specific to our rabbit experiment.

In conclusion, our findings suggest that early Scl-Ab treatment inhibited the adipogenic effect of estrogen deficiency in terms of restoring marrow fat expansion seen in a rabbit model of postmenopausal osteoporosis, in addition to its role in suppressing bone resorption and promoting bone formation. MR spectroscopy appears be a useful tool for longitudinal and interventional assessments in osteoporosis-related fields.

## Data Availability

The datasets for this manuscript are not publicly available because Confidentiality and integrity may be required. Requests to access the datasets should be directed to SL, MD Department of Radiology, The Second Military Medical University Affiliated Gongli Hospital, No. 219 Miaopu Road, Shanghai 200135, China. Email: ppmmri@163.com.

## Author Contributions

SL: concept of study design, data acquisition, data postprocessing, statistical analysis, and drafting of manuscript. BH: data post-processing and critical revision of manuscript. BJ: data acquisition, data post-processing, and critical revision of manuscript. MG: subject recruitment, data acquisition, and critical revision of manuscript, subject recruitment, data acquisition, and critical revision of manuscript. XY: concept of study design, subject recruitment, and critical revision of manuscript, data post-processing and critical revision of manuscript. YY: concept of study design, statistical analysis, and critical revision of manuscript, data acquisition and critical revision of manuscript.

### Conflict of Interest Statement

The authors declare that the research was conducted in the absence of any commercial or financial relationships that could be construed as a potential conflict of interest.
